# *QuickStats:* Percentage* of Adults Aged ≥18 Years Who Felt Worried, Nervous, or Anxious Daily or Weekly,^†^ by Age Group and Employment Status^§^ — National Health Interview Survey,^¶^ United States, 2017

**DOI:** 10.15585/mmwr.mm6816a5

**Published:** 2019-04-26

**Figure:**
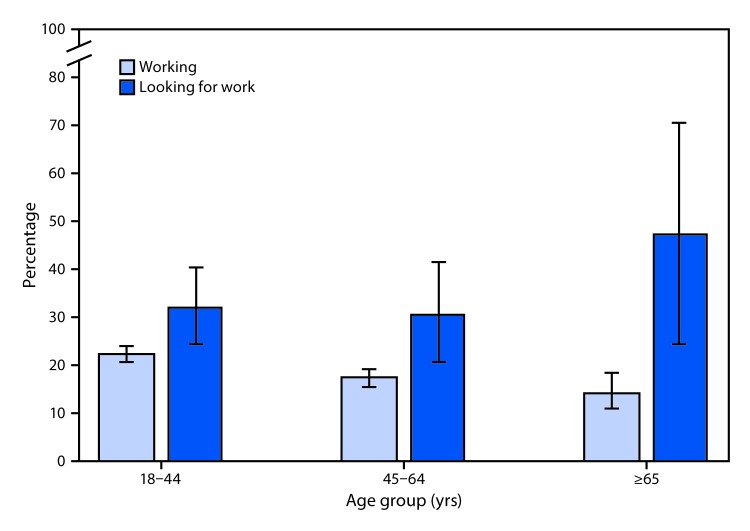
In 2017, compared with adults currently working, the percentage of adults who reported feeling worried, nervous, or anxious daily or weekly was higher among those looking for work in all three age groups: 18–44 years (22.4% versus 32.1%), 45–64 years (17.3% versus 30.4%), and ≥65 years (14.3% versus 47.2%). The percentage of currently working adults who reported feeling worried, nervous, or anxious declined with age.

**Source:** National Health Interview Survey, 2017. https://www.cdc.gov/nchs/nhis.htm.

